# Association between composite dietary antioxidant index and hyperlipidemia: a cross-sectional study from NHANES (2005–2020)

**DOI:** 10.1038/s41598-024-66922-0

**Published:** 2024-07-10

**Authors:** Minli Zhao, Danwei Zhang, Qiuping Zhang, Yuan Lin, Hua Cao

**Affiliations:** 1 Fujian Children’s Hospital (Fujian Branch of Shanghai Children’s Medical Center), College of Clinical Medicine for Obstetrics & Gynecology and Pediatrics, Fujian Medical University, Fuzhou, 350014 China; 2 Shengli Clinical Medical College of Fujian Medical University, Fuzhou, 350001 China; 3 Fujian Medical University, University Town, 1 Xue Yuan Road, Fuzhou, 350122 China

**Keywords:** Composite dietary antioxidant index, Hyperlipidemia, Oxidative stress, NHANES, Cardiology, Diseases, Endocrinology, Health care, Medical research, Risk factors

## Abstract

The association between the composite dietary antioxidant index (CDAI) and hyperlipidemia remains unclear. Therefore, this study aimed to investigate the relationship between CDAI and hyperlipidemia. The data used in this study were obtained from the National Health and Nutrition Examination Survey (NHANES) dataset spanning from 2005 to 2020. Based on 24-h dietary recall interviews, the CDAI was calculated using the intake of six dietary antioxidants. Univariate and multivariate logistic regression models were employed to investigate the relationship between CDAI and the occurrence of hyperlipidemia. Additionally, restricted cubic spline (RCS) analysis was utilized to investigate potential non-linear relationships between the CDAI and risk of hyperlipidemia. The final analysis included 30,788 adults in the United States, among whom 25,525 (82.91%) were diagnosed with hyperlipidemia. A significant negative correlation was observed between the CDAI and hyperlipidemia in the unadjusted (Odds ratio [OR] 0.97 [95% CI 0.96, 0.98]) and multi-variable adjusted (OR 0.98 [95% CI 0.97, 0.99]) models. When the CDAI values were analyzed as a categorical variable, individuals in the highest quartile (OR 0.82 [95% CI 0.73, 0.92]) exhibited a nearly one fifth decreased risk of hyperlipidemia compared to those in the lowest quartile. Additionally, RCS analysis revealed a linear relationship between CDAI and hyperlipidemia (*P* for nonlinearity = 0.124). The results remained consistent across subgroups except for individuals under the age of 60 or those with diabetes mellitus. There was a significant negative correlation between the CDAI and risk of hyperlipidemia, indicating that maintaining an optimal CDAI level could effectively reduce the incidence of hyperlipidemia.

## Introduction

Hyperlipidemia is a metabolic disorder characterized by excessively elevated serum lipid levels^1^, which can be classified into different clinical types including hypercholesterolemia, hypertriglyceridemia, mixed hyperlipidemia, and high-density lipoproteinemia^2^. Hyperlipidemia has emerged as an important public health problem with increased prevalence worldwide. In the US, it is estimated that around 25 million adults had total cholesterol (TC) levels exceeding 200 mg/dL between 2017 and 2020^3^. Hyperlipidemia was not only a major modifiable risk factor for cardiovascular and cerebrovascular diseases^4^ but also a leading cause of death^5^. Beyond pharmaceutical treatments, emerging studies emphasize the impact of non-pharmacological interventions in managing hyperlipidemia^6^.

Previous evidence suggested that hyperlipidemia was primarily associated with increased oxidative stress throughout the body^7–9^. Oxidative stress is a physiological condition characterized by an imbalance in the oxidation–reduction status within the cells or entire body, leading to over production of reactive oxygen species (ROS) such as oxygen free radicals^10^. The ROS cause damage to biomolecules within cells, such as lipids, proteins, and DNA, resulting in cell injury and abnormal inflammation^11^. Studies have shown that increasing antioxidant intake in the diet could reduce oxidative stress levels in the body^12^. Therefore, we speculated that modifying the dietary structure might treat hyperlipidemia by reducing oxidative stress. This hypothesis was first confirmed in a cross-sectional study from Korea, where they found that dietary total antioxidant capacity (TAC) was associated with dyslipidemia (hypercholesterolemia, hypertriglyceridemia, and hypoHDL-cholesterolemia)^13^. The Composite Dietary Antioxidant Index (CDAI) was a nutritional tool developed to assess the overall antioxidant capacity of an individual's daily food intake^14–17^. It took into account six specific antioxidants, namely vitamin A, vitamin C, vitamin E, carotenoids, selenium, and zinc^14–17^, with a higher CDAI score indicating a dietary pattern rich in antioxidants. Though previous studies have confirmed the relationship between CDAI and several chronic diseases, hypertension^18^, diabetes mellitus^19^, coronary heart disease^20^. However, the association between CDAI and hyperlipidemia has not been elucidated.

This study intended to determine the relationship between CDAI and hyperlipidemia by using data from the National Health and Nutrition Examination Survey (NHANES), thus, to provide better dietary guidance for hyperlipidemia management.

## Methods

### Data source and population

**Figure 1:**
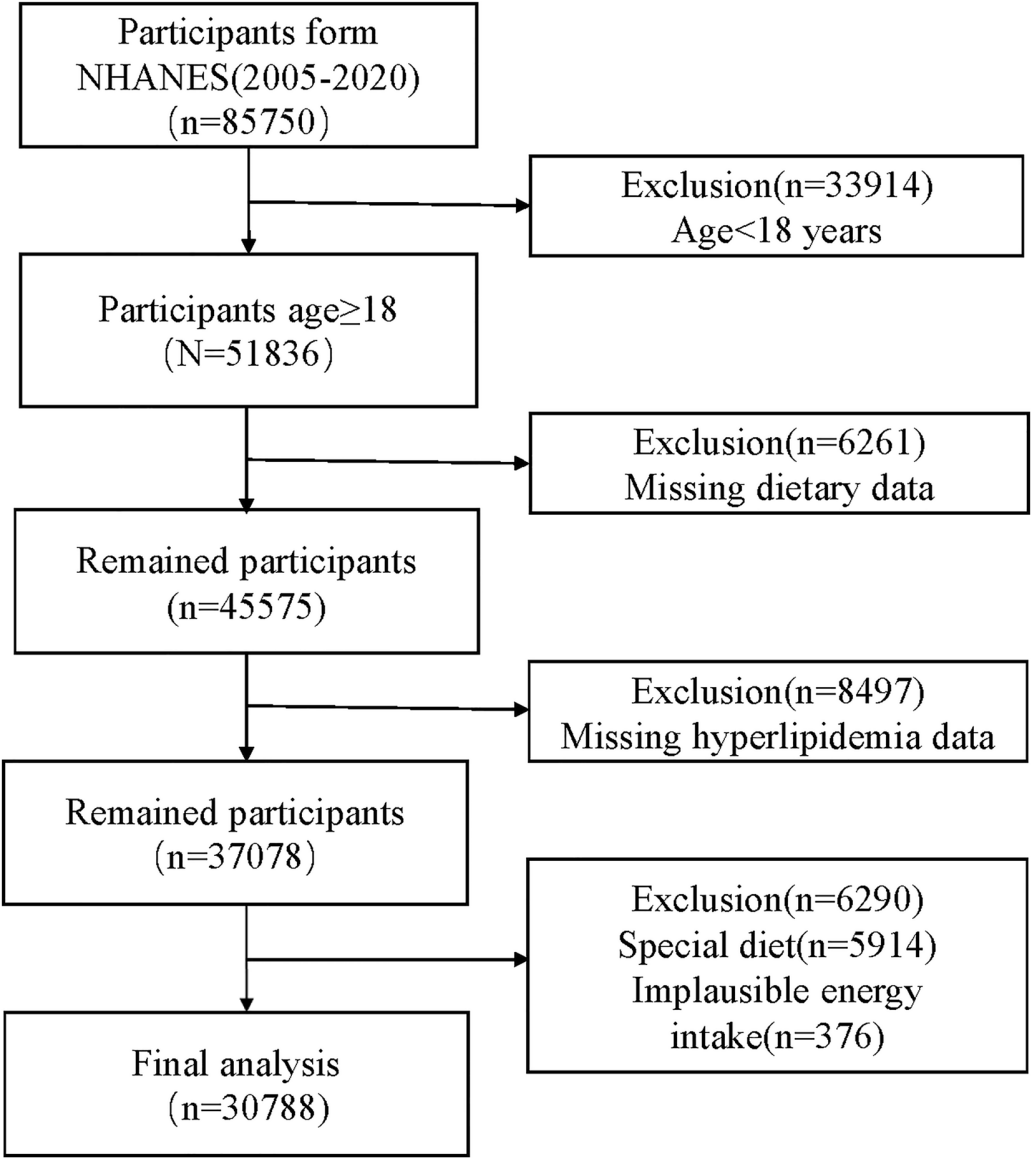
The flow chart of study participant selection.

The data used in this study were obtained from the NHANES 2005–2020, a comprehensive cross-sectional survey that assessed the nutritional status and health of the US population^21,22^. Detailed information about the design methods and contents of NHANES could be found at http://www.cdc.gov/nchs/nhanes.htm. The study protocols were approved by the Ethics Review Board of the National Center for Health Statistics, and written informed consent was obtained from all participants prior to their participation in the survey. To reduce sampling and recall bias, this study gathered data for all participants (n = 85,750) who had undergone two dietary recalls from the NHANES datasets. This study initially excluded participants (n = 33,914) who were below the age of 18. Subsequently, participants lacking dietary information (n = 6261) and those without hyperlipidemia data (n = 8497) were also excluded. Moreover, this study also excluded participants with special diet (n = 5914) and implausible energy intake (< 500 kcal/day or ≥ 5000 kcal/day) (n = 376)^13^. The process of participant selection is depicted in Fig. 1, and the final analysis included 30,788 eligible participants.

### Definition of CDAI

The information regarding dietary antioxidant intake was obtained through two separate 24-h dietary recall interviews^23^. Trained dietary surveyors recorded detailed information about the participants' dietary consumption in the 24 h prior to the interview. The first dietary recall was conducted through face-to-face interviews, while the second recall was carried out via telephone consultation 3–10 days later. Utilizing the average dietary intake data from two non-consecutive days was considered more accurate than relying solely on data from a single day^16,24^. Six antioxidants (zinc, selenium, total carotenoids, vitamins A, C, and E) were standardized by subtracting the mean and dividing by the standard deviation (SD). The CDAI, developed by Wright et al.^25^, was based on the sum of these standardized consumptions^17,26^. It should be noted that the calculation of dietary antioxidant intake in this study did not include antioxidants obtained from supplements, medications, or other additional sources. The detailed calculation formula is presented below:

$${\text{CDAI}} = \mathop \sum \limits_{n = 1}^{6} \left( {{ }\frac{Individual\;Intake - Mean}{{SD}}} \right).$$

### Definition of hyperlipidemia

The hyperlipidemia status was evaluated based on the Adult Treatment Panel III (ATP 3) guidelines of the National Cholesterol Education Program (NCEP) for adults^27^. A diagnosis of hyperlipidemia was confirmed if any of the following five conditions were met: triglycerides (TG) ≥ 150 mg/dL, total cholesterol (TC) ≥ 200 mg/dL, low-density lipoprotein (LDL) ≥ 130 mg/dL, or high-density lipoprotein (HDL) ≤ 40 mg/dL in males and ≤ 50 mg/dL in females^27^. Additionally, participants who reported using cholesterol-lowering medications were also classified as having hyperlipidemia^28,29^.

### Covariates

To evaluate the potential impact of confounding factors, we carefully selected several covariates, including age, body mass index (BMI), gender, race, education level, poverty income ratio (PIR), marital status, alcohol consumption, smoking status, diabetes mellitus, hypertension, physical activity and energy intake. The age of the participants selected for this study refers to their age at the time of screening. BMI was calculated by dividing an individual's weight (in kilograms) by the square of their height (in square meters). Gender was categorized as male or female. Race was classified as non-Hispanic White, non-Hispanic Black, Mexican American, other Hispanic, and other race. Education level was grouped as less than high school, high school, or college and above. The household socioeconomic status, estimated using the PIR index, was categorized into three groups: low (< 1.5), middle (1.5–3.5), and high (> 3.5)^30^. The marital status categories were defined as single, which included individuals who were separated, widowed, never married, or divorced, and married^31^. Alcohol consumption was classified into three categories: mild, moderate, and heavy. Heavy alcohol consumption was defined as consuming ≥ 3 drinks per day for females or ≥ 4 drinks per day for males, or engaging in binge drinking on five or more days per month. Moderate alcohol consumption was defined as consuming ≥ 2 drinks per day for females and ≥ 3 drinks per day for males, or engaging in binge drinking on ≥ 2 days per month. Mild alcohol consumption was considered as other forms of alcohol intake^17,28,32^. Smoking status was classified into three categories: never, former, and current. Never smokers were individuals who had smoked fewer than 100 cigarettes in their lifetime. Former smokers had smoked more than 100 cigarettes in their lifetime but currently did not smoke at all. Current smokers had smoked more than 100 cigarettes in their lifetime and currently smoked either occasionally or daily^16,28^. Diabetes mellitus was defined as individuals diagnosed with diabetes, those using anti-diabetes drugs or insulin. Hypertension was defined as individuals with an average blood pressure exceeding 140/90 mmHg, self-reporting a physician-diagnosed hypertension, or currently taking medication for hypertension. Physical activity was defined as total MET minutes per week, calculated by multiplying the weekly volume of physical activity (duration × frequency) for each activity by its corresponding MET value^16,33^. Energy intake was calculated by averaging the dietary recall data collected over a span of two days.

### Statistical analysis

Participants characteristics were compared between participants with and without hyperlipidemia. Continuous data were presented as means (± SD) or interquartile range [IQR], and compared using either t test or non-parametric test. Categorical variables were presented as percentages (%) and compared using the chi-square test. Multiple imputation was used to handle missing values in the dataset. Univariate and multivariate logistic regression models were used to examine the relationship between CDAI (including both continuous variables and quartile groups) and hyperlipidemia, with the covariates listed above. In Model 1, no covariate was adjusted. In Model 2, adjustments were made for age and gender. Model 3 included additional covariates such as BMI, race, education level, PIR, marital status, alcohol consumption, smoking status, diabetes mellitus, hypertension, physical activity, and energy intake. Restricted cubic splines (RCS) were also used to explore potential nonlinearity. Identical analysis approach was employed to examine specific subgroups, including age, gender, BMI, hypertension and diabetes mellitus. All statistical analyses were performed using SPSS 27 and R 4.2.2 software. Results with a *P* < 0.05 were considered statistically significant.

### Ethics statement

The studies were approved by The Research Ethics Review Board at the National Center for Health Statistics (NCHS). The participants provided their written informed consent to participate in this study.

## Results

### Baseline characteristics

In the final analysis, a total of 30,388 individuals from the NHANES datasets were included, with an average age of 49.17 (± 18.54) years, of whom 50.85% were female. There were several characteristics of significant differences between hyperlipidemic and non-hyperlipidemic population (Table 1). Compared to individuals without hyperlipidemia, those with hyperlipidemia exhibited several characteristics: older age, higher BMI levels, greater proportion of female gender, non-Hispanic white ethnicity, college education or above, married status, mild alcohol consumption, non-smoking history, absence of diabetes or hypertension, lower energy intake and physical activity levels, and higher CDAI levels (all *P* < 0.05).

**Table 1:** Demographic and other features of participants based on the presence of hyperlipidemia from the NHANES 2005–2020.

Variables	Overall	Non-hyperlipidemia	Hyperlipidemia	*P* value
	(n = 30,788)	(n = 5263)	(n = 25,525)	
Age	49.17 (± 18.54)	40.12 (± 18.36)	51.03 (± 18.02)	0.000
BMI (kg/m^2^)	29.16 (± 6.82)	26.44 (± 6.51)	39.72 (± 6.75)	< 0.001
Gender				< 0.001
Male	15,132 (49.15)	2727 (51.81)	12,405 (48.60)	
Female	15,656 (50.85)	2536 (48.19)	13,120 (51.40)	
Race				< 0.001
Non-Hispanic White	12,823 (41.65)	1969 (37.41)	10,854 (42.52)	
Non-Hispanic Black	6548 (21.27)	1431 (27.19)	5117 (20.05)	
Mexican American	4958 (16.10)	788 (14.97)	4170 (16.34)	
Other Hispanic	3001 (9.75)	429 (0.82)	2572 (10.08)	
Other races	3458 (11.23)	646 (12.27)	2812 (11.02)	
Education levels				< 0.001
Less than high school	7714 (25.06)	1145 (21.76)	6569 (25.74)	
High school or equivalent	7529 (24.45)	1203 (22.86)	6326 (24.78)	
College or above	15,545 (50.49)	2915 (55.39)	12,630 (49.48)	
Poverty income ratio				0.34
Low	11,729 (38.10)	2043 (38.82)	9686 (37.95)	
Middle	9997 (32.47)	1712 (32.53)	8285 (32.46)	
High	9062 (29.43)	1508 (28.65)	7554 (29.59)	
Marital status				< 0.001
Single	12,358 (40.14)	2427 (46.11)	9931 (38.91)	
Married	18,430 (59.86)	2836 (53.89)	15,594 (61.09)	
Alcohol consumption				< 0.001
Mild	19,578 (63.59)	3023 (57.44)	16,555 (64.86)	
Moderate	5021 (16.31)	989 (18.79)	4032 (15.80)	
Heavy	6189 (20.10)	1251 (23.77)	4938 (19.35)	
Smoking status				< 0.001
Never	17,047 (55.37)	3174 (60.31)	13,873 (54.35)	
Former	7363 (23.92)	1049 (19.93)	6314 (24.74)	
Now	6378 (20.72)	1040 (19.76)	5338 (20.91)	
Diabetes mellitus				< 0.001
No	27,072 (87.93)	4990 (94.81)	22,082 (86.51)	
Yes	3716 (12.07)	273 (5.19)	3443 (13.49)	
Hypertension				< 0.001
No	17,903 (58.15)	3978 (75.58)	13,925 (54.55)	
Yes	12,885 (41.85)	1285 (24.42)	11,600 (45.45)	
Physical activity, MET-min/week	2160.00 (720.00, 6000.00)	2760.00 (960.00, 7200.00)	1960.00 (720.00, 5760.00)	< 0.001
Energy intake (kcal/day)	1924.00 (1469.50, 2487.50)	2037.50 (1557.00,2606.00)	1901.50 (1452.50, 2458.50)	< 0.001
CDAI	− 0.61 (− 2.68, 1.94)	− 2.00 (− 2.37, 2.48)	− 2.74 (− 6.91, 1.82)	< 0.001

Data are presented as mean (SD) or n (%).

*BMI* body mass index, *CDAI* composite dietary antioxidant index, *PIR* Ratio of family income to poverty.

### Associations between CDAI levels and risks of hyperlipidemia

**Figure 2:**
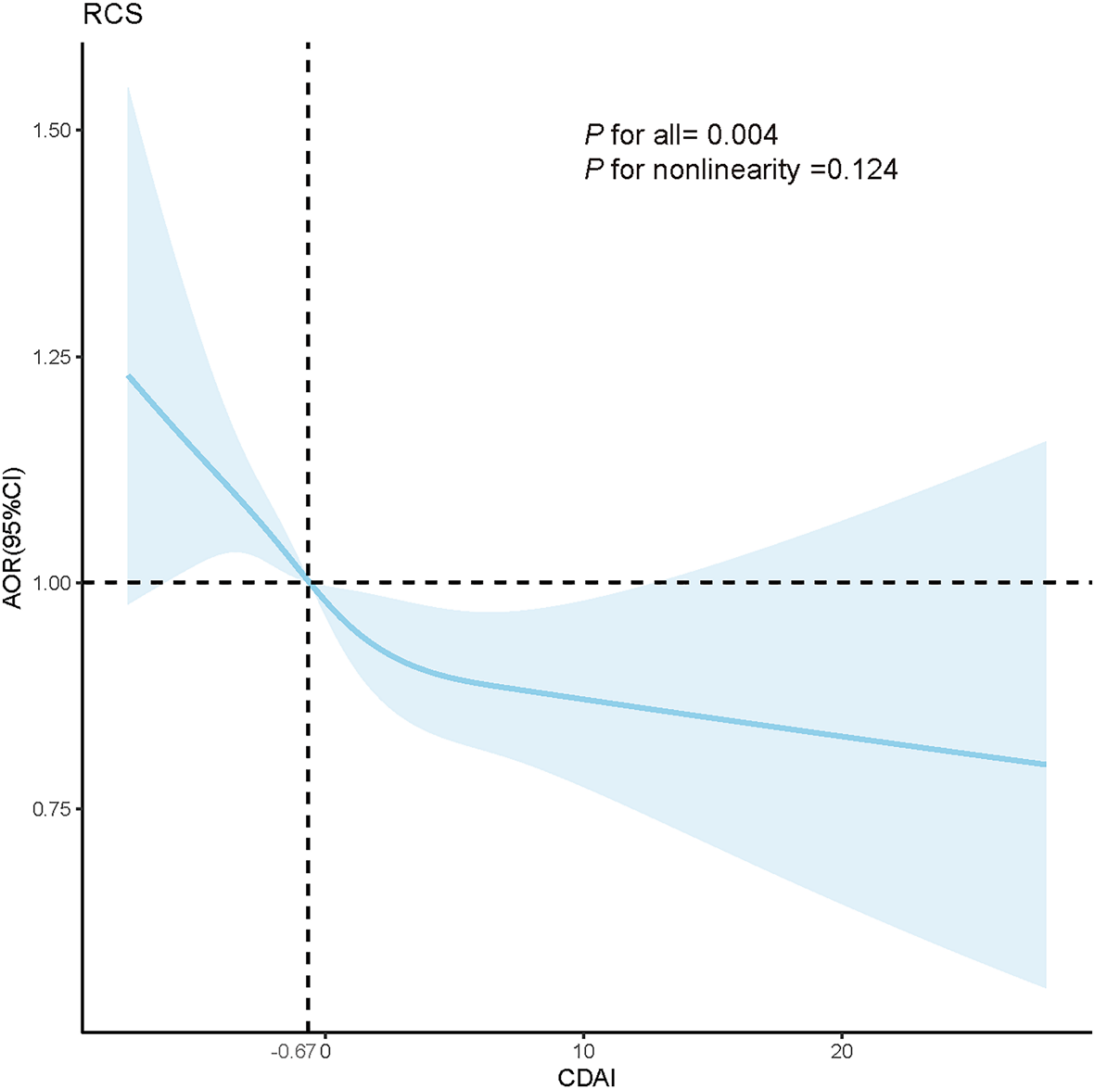
The dose–response relationship between CDAI and the prevalence of hyperlipidemia. The blue line represents AOR, and the blue transparent area represents 95% CI. AOR results are adjusted based on Model 3. Abbreviations: AOR, adjusted odds ratio; CI, confidence interval, CDAI, composite dietary antioxidant index.

Logistic regression models were used to explore the associations between CDAI and hyperlipidemia (Table 2). In Model 1 (unadjusted), a negative association was observed between continuous CDAI and hyperlipidemia (OR 0.97 [95% CI 0.96, 0.98]). This relationship remained consistent after adjusting for covariates in Model 2 (OR 0.98 [95% CI 0.97, 0.98]) and Model 3 (OR 0.98 [95% CI 0.97, 0.99]). Compared to the CDAI of the lowest quartiles, participants in the third quartile (Q3) (OR 0.89 [95% CI 0.80, 0.98]) and fourth quartile (Q4) (OR 0.82 [95% CI 0.73, 0.92]) of CDAI had significantly decreased risk of hyperlipidemia, with a significant trend of the quartiles (*P* for trend: 0.01). The RCS analysis confirmed a linear relationship between CDAI and the risk of hyperlipidemia after full adjustment (*P* for nonlinearity: 0.124, Fig. 2).

**Table 2:** Association of composite dietary antioxidant index and hyperlipidemia.

Characteristics	Model 1		Model 2		Model 3	
	OR (95% CI)	*P*	OR (95% CI)	*P*	OR (95% CI)	*P*
Continuous	0.97 (0.96,0.98)	< 0.001	0.98 (0.97,0.98)	< 0.001	0.98 (0,97,0.99)	< 0.001
Quartile						
Q1	Ref	Ref	Ref	Ref	Ref	Ref
Q2	0.93 (0.85,1.01)	0.091	0.93 (0.85,1.02)	0.136	0.94 (0.85,1.03)	0.197
Q3	0.84 (0.78,0.91)	< 0.001	0.88 (0.80,0.96)	0.004	0.89 (0.80,0.98)	0.022
Q4	0.72 (0.66,0.78)	< 0.001	0.78 (0.71,0.85)	< 0.001	0.82 (0.73,0.92)	< 0.001
*P* for trend	–	< 0.001	–	< 0.001	–	0.010

Model 1 was not adjusted; Model 2 was adjusted for age and gender; Model 3 was adjusted for age, gender, BMI, race, education levels, poverty income ratio, marital status, alcohol consumption, smoking status, diabetes mellitus, hypertension, physical activity, and energy intake.

*OR* odds ratio, *CI* confidence interval, *CDAI* composite dietary antioxidant index.

### Association between six antioxidants (components of CDAI) and hyperlipidemia

Table 3 indicates the relationship between individual dietary antioxidant and hyperlipidemia. After adjustment, it was observed that dietary intake of vitamin E (OR 0.91 [95% CI 0.88, 0.94]), selenium (OR 0.95 [95% CI 0.91, 0.99]), and carotenoids (OR 0.96 [95% CI 0.93, 0.98]) exhibited a negative association with the risk of hyperlipidemia. However, a higher intake of dietary vitamin A, vitamin C and zinc did not show a decreased risk of hyperlipidemia.

**Table 3:** Association of six components of composite dietary antioxidant index and hyperlipidemia.

	Model 1		Model 2		Model 3	
	OR (95%CI)	*P*	OR (95%CI)	*P*	OR (95%CI)	*P*
Components						
Vitamin A	0.98 (0.95, 1.01)	0.107	0.94 (0.91, 0.96)	< 0.001	0.97 (0.94,1.00)	0.053
Vitamin C	0.94 (0.91, 0.96)	< 0.001	0.94 (0.92, 0.97)	< 0.001	0.99 (0.96,1.02)	0.466
Vitamin E	0.88 (0.86, 0.91)	< 0.001	0.89 (0.87, 0.92)	< 0.001	0.91 (0.88,0.94)	< 0.001
Zine	0.93 (0.91, 0.96)	< 0.001	0.99 (0.96, 1.02)	0.547	1.02 (0.98,1.06)	0.403
Selenium	0.87 (0.85, 0.90)	< 0.001	0.95 (0.92, 0.98)	0.002	0.95 (0.91,0.99)	0.015
Carotenoid	0.93 (0.91, 0.96)	< 0.001	0.93 (0.90, 0.96)	< 0.001	0.96 (0.93,0.98)	0.003

Model 1 was not adjusted; Model 2 was adjusted for age and gender;

Model 3 was adjusted for age, gender, BMI, race, education levels, poverty income ratio, marital status, alcohol consumption, smoking status, diabetes mellitus, hypertension, physical activity, and energy intake.

*OR* odds ratio, *CI* confidence interval, *CDAI* composite dietary antioxidant index.

### Subgroup analysis

**Figure 3:**
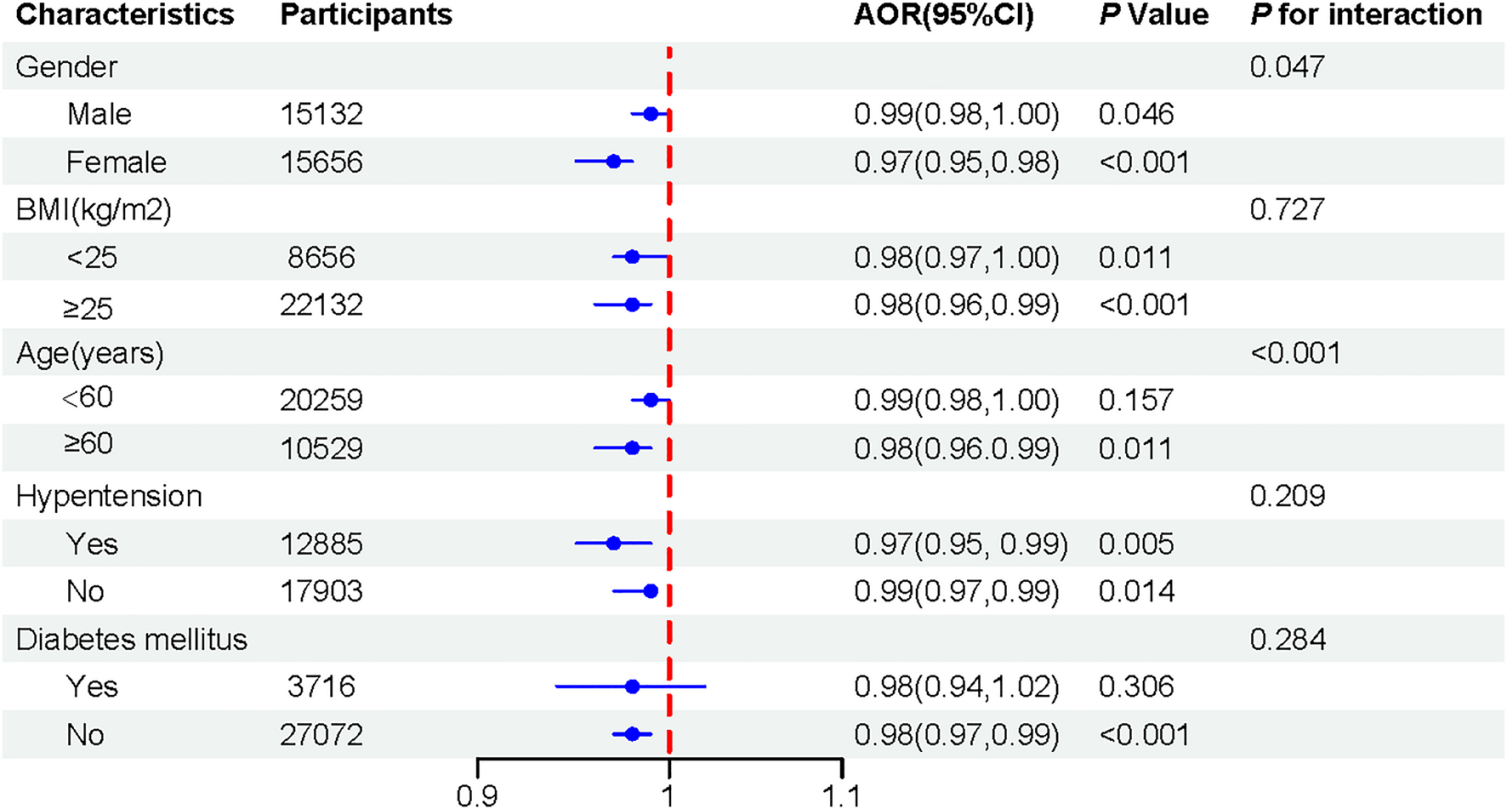
Subgroup analysis of the relationship between CDAI and hyperlipidemia. The outcome is adjusted for all covariates, with the exception of the corresponding stratification variable. Abbreviations: AOR, adjusted odds ratio; CI, confidence interval, BMI, body mass index.

In all the subgroups (Fig. 3), consistently negative associations were observed between CDAI and hyperlipidemia after adjustment, though the odds in the subgroups of age and diabetes mellitus were insignificant. The *P* values for interaction were significant only in subgroups stratified by age (*P* for interaction: < 0.001) and gender (*P* for interaction: 0.047). Significant association was found only in subgroup populations with age greater than 60 years (OR 0.98 [95% CI 0.96, 0.99]), and the effect of CDAI was greater in the female population (OR 0.97 [95% CI 0.95, 0.98]) than the males (OR 0.99 [95% CI 0.98, 1.00]).

## Discussion

In this study, involving 30,788 participants in the final analysis, we observed that elevated CDAI was significantly associated with a lower risk of hyperlipidemia, suggesting that improving CDAI might play a protective role in the development of hyperlipidemia. This study also showed that higher levels of vitamin E, selenium, and carotenoids were associated with a lower incidence of hyperlipidemia, while no significant correlation was observed in vitamin A, vitamin C, and zinc. The results remained consistent across most prespecified subgroups, except for individuals under the age of 60 or those with diabetes mellitus.

The CDAI, a widely recognized scoring system for total dietary antioxidants, has been extensively utilized in clinical research. For example, several cross-sectional studies have investigated and established the correlation between CDAI and various health conditions, such as coronary heart disease^20^, hypertension^18^, stroke^15^, diabetes mellitus^19^. While research on the association between CDAI and hyperlipidemia is still relatively limited, the exploration of using specific dietary antioxidants for managing hyperlipidemia has become a highly debated topic^13^. The mechanisms responsible for the negative correlation between CDAI and risk of hyperlipidemia are not yet well understood. It is known that oxidative stress plays a crucial role in the development of hyperlipidemia. In reverse, high levels of blood lipid typically result in increased levels of oxidative stress, which in turn, can worsen the progression of hyperlipidemia^34^.

The CDAI consists of six antioxidant components including antioxidant vitamins (vitamin A, vitamin C, and vitamin E) and micronutrients (zinc, selenium, and carotenoids), all of which play a crucial role in combating oxidative stress^35,36^. Vitamins play an essential role in lipid metabolism reactions^37^. Marguerite et al. conducted a randomized controlled study demonstrating that supplementation with antioxidant vitamins A, C and E could restore endothelial function in individuals with hyperlipidemia, thereby improving their long-term cardiovascular health^38^. However, this study found no association between vitamins A, C and hyperlipidemia. The difference in results may be attributed to variations in dietary habits across different populations. Consistent with our findings, a systematic review by Li et al. confirmed that serum zinc was not irrelevant with hyperlipidemia^39,40^. A randomized trial found that taking selenium supplementation could decrease non-high-density lipoprotein cholesterol levels^41^. However, there were also epidemiological studies indicating a positive association between selenium and lipid levels^37,42,43^. This difference could be attributed to several reasons. The definition of hyperlipidemia in this study was comprehensive and considered different types of blood lipids, including TC, TG, HDL, and LDL^44^. Furthermore, variances in the regions, races, and eating habits of the study population could also account for the diverse results. For carotenoids, many studies found a protective effect of carotenoids on cardiovascular diseases^40,45^, which was consistent with our findings. Vitamins E play a crucial role in alleviating oxidative stress and protecting cells from oxidative damage^44^. Selenium binds to selenoproteins, thereby preventing lipid peroxidation and reducing oxidative damage to cells^46^. Carotenoids exert antioxidative roles through both direct interactions with free radicals via electron or hydrogen atom transfer^47^ and by activating the hormetic-dependent activation of vitagenes^48^. Hence, we hypothesized that CDAI could potentially prevent the onset of hyperlipidemia induced by oxidative stress.

Subgroup analysis revealed that the negative association between CDAI and hyperlipidemia was robust across gender, BMI, and hypertension, proving the reliability and applicability of our findings. Notably, no association was found between CDAI and hyperlipidemia among individuals under 60 years old or those with diabetes mellitus. It was speculated that the beneficial effect of CDAI on hyperlipidemia may have been weakened in diabetic mellitus patients due to the control of a diabetic diet as well as the administration of insulin and hypoglycemic drugs. However, the exact mechanism remained unclear and required further investigation. Additionally, this study suggested that the association between CDAI and risk of hyperlipidemia was influenced by gender and age. Further research was warranted to investigate these mechanisms and gain a deeper understanding of this intricate relationship.

The CDAI has served as a gauge for a food's capacity to combat oxidative stress and has garnered widespread usage. By evaluating the quantity of antioxidants within food and their effectiveness in neutralizing oxidative substances, it assists in appraising the nutritional worth of various food items. Consequently, it empowers individuals to make healthier dietary selections, thereby preserving optimal health. The results of this study provide important support for the development of healthier dietary guidelines, assisting people in making better food choices to maintain cardiovascular health. Additionally, it may also contribute to deepening our understanding of antioxidants and their mechanisms of action in the human body, offering further insights for future research and clinical practice.

### Strengths and limitations

The strengths of this study are as follows: Firstly, the data utilized in this study were derived from the NHANES dataset, which is of high quality and has garnered widespread recognition and application. Secondly, this study was the first to investigate the relationship between CDAI and hyperlipidemia in such a large population, as well as in specific subgroups. The limitations of our study can be summarized as follows: Firstly, although confounding factors have been extensively adjusted, there are still other potential factors that cannot be completely ruled out. Secondly, given the lack of direct correlation between the intake of nutrients of this study and the intake of other nutrients in this study, we did not adjust for them as confounding factors. More research is needed in the future to elucidate whether other nutrients may act as confounders in exploring the association between CDAI and risk of hyperlipidemia. Thirdly, the cross-sectional design in this study limited our ability to explore the causal relationships.

## Conclusions

This cross-sectional study demonstrated that higher CDAI was significantly associated with a lower risk of hyperlipidemia. Moreover, the association was also found in individual components as vitamin E, selenium, and carotenoids. Improving dietary antioxidants consumption could be regarded as a practical strategy in hyperlipidemia prevention and lipid management.

## Data Availability

The detailed datasets about the surveys are available at www.cdc.gov/nchs/nhanes.
